# Low serum total CO_2_ and its association with mortality in patients being followed up in the nephrology outpatients clinic

**DOI:** 10.1038/s41598-021-81332-2

**Published:** 2021-01-18

**Authors:** Kyung Don Yoo, Jung Nam An, Yong Chul Kim, Jeonghwan Lee, Kwon-Wook Joo, Yun Kyu Oh, Yon Su Kim, Chun Soo Lim, Sohee Oh, Jung Pyo Lee

**Affiliations:** 1 Department of Internal Medicine, Ulsan University Hospital, University of Ulsan College of Medicine, Ulsan, Korea; 2 Department of Internal Medicine, Hallym University Sacred Heart Hospital, Anyang, Gyeonggi-do Korea; 3 Department of Internal Medicine, Seoul National University Hospital, Seoul, Korea; 4 Department of Internal Medicine, Seoul National University Boramae Medical Center, 20, Boramae-ro 5-gil, Dongjak-gu, Seoul, 07061 Korea; 5 Department of Internal Medicine, Seoul National University College of Medicine, Seoul, Korea; 6 Department of Biostatistics, Seoul National University Boramae Medical Center, 20, Boramae-ro 5-gil, Dongjak-gu, Seoul, 07061 Korea

**Keywords:** Health care, Medical research, Nephrology, Risk factors

## Abstract

Large-scale studies have not been conducted to assess whether serum hypobicarbonatemia increases the risk for kidney function deterioration and mortality among East-Asians. We aimed to determine the association between serum total CO_2_ (TCO_2_) concentrations measured at the first outpatient visit and clinical outcomes. In this multicenter cohort study, a total of 42,231 adult nephrology outpatients from 2001 to 2016 were included. End-stage renal disease (ESRD) patients on dialysis within 3 months of the first visit were excluded. Instrumental variable (IV) was used to define regions based on the proportion of patients with serum TCO_2_ < 22 mEq/L. The crude mortality rate was 12.2% during a median 77.0-month follow-up period. The Cox-proportional hazard regression model adjusted for initial kidney function, alkali supplementation, and the use of diuretics demonstrated that low TCO_2_ concentration was not associated with progression to ESRD, but significantly increased the risk of death. The IV analysis also confirmed a significant association between initial TCO_2_ concentration and mortality (HR 0.56; 95% CI 0.49–0.64). This result was consistently significant regardless of the underlying renal function. In conclusion, low TCO_2_ levels are significantly associated with mortality but not with progression to ESRD in patients with ambulatory care.

## Introduction

Serum hypobicarbonatemia was observed in about 20% of patients with chronic kidney disease (CKD) stages 4–5 in the NHANES cohort^1^ and in 26.0% and 47.4% of patients with CKD stages 4 and 5, respectively, in South Korea^2,3^. Previous epidemiologic cohort studies showed that CKD patients with higher serum bicarbonate levels had lower risks of developing end-stage renal disease (ESRD) in various populations^4–6^. Several observational studies involving patients with CKD found that lower total serum CO_2_ (TCO_2_) levels, higher net endogenous acid production^7^, and high dietary acid loads^8,9^ were associated with higher risks of progressive renal function deterioration among various ethnic groups^7–11^. Reaven et al. recently conducted a well-designed observational study which included the medical records of > 50,000 community-based patients with advanced CKD (stages 3a-5) obtained from a diverse geographically distributed database^12^. The longitudinal study analyzed the10-year data of this large US community-based population to assess the effects of hypobicarbonatemia on mortality and kidney outcomes. The authors found that death, ESRD with ongoing dialysis, and eGFR reduction of ≥ 40% were significantly higher among patients with hypobicarbonatemia compared to those with normal serum bicarbonate levels (*P* < 0.001 for all results, regardless of age group)^12^. The prevalence of early CKD is increasing in South Korea, and data on the role of asymptomatic metabolic acidosis in CKD progression is limited^13–15^.

Notably, it is difficult to evaluate acid-base status in an outpatient setting because of the difficulty associated with performing arterial blood gas analysis in this setting. In this study, we investigated the long-term clinical impact of hypobicarbonatemia on the development of incident ESRD and overall mortality in East-Asians, using the level of TCO_2_ on venous sampling. We attempted to determine the effects of hypobicarbonatemia in a retrospective cohort from the patients with various regions of South Korea, using instrumental variable (IV) analysis.

## Methods

### Study subjects

This was a retrospective observational cohort study of patients tracked in the outpatient nephrology clinic of two tertiary hospitals in South Korea (Seoul National University Hospital and Seoul National University Boramae Hospital). Hospital administrative data were extracted, and the 10^th^ International Code of Disease Classification (ICD-10) was used. Data were collected using electronic health records following the methods also described in previous studies^16^. In detail, the date of birth, sex, age, body mass index (kg/m^2^), and data related to other comorbidities (using the diagnostic code by ICD-10) and medication were recorded.

**Figure 1:**
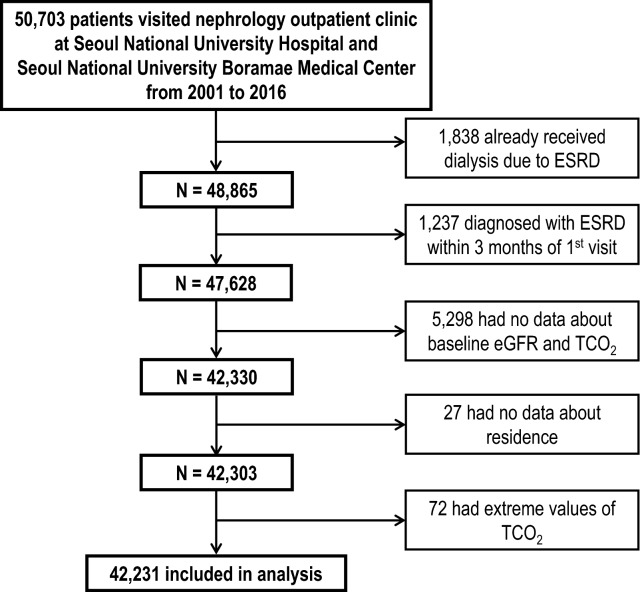
Study flow chart.

A total of 50,703 patients were examined from January 1, 2001, to December 31, 2016. Of the total 50,703 patients, we excluded 1,838 patients who had already been on dialysis with ESRD and 1,237 patients with acute kidney injury requiring renal replacement therapy who had been on dialysis within three months of hospital visit. Patients without record of serum creatinine levels or total CO_2_ (TCO_2_) (n = 5,298), patients with no address (n = 27), and those with extreme values of TCO_2_ (n = 72) were also excluded. The final analysis was performed including 42,231 patients (Fig. 1).

### Data collection

The Low TCO_2_ group was defined as TCO_2_ < 22 mEq/L. Among the drugs used, medications potentially affecting the status of TCO_2_ level, data of patients who had a prescription of sodium bicarbonate, renin–angiotensin–aldosterone system (RAAS) blockades, including angiotensin-converting-enzyme inhibitors (ACE inhibitors, ACEi) and angiotensin II receptor blockers (ARBs), and diuretics were identified. Diuretics group included acetazolamide, spironolactone, amiloride, triamterene, furosemide, torasemide, thiazide, indapamide and metolazone.

We included only those patients who had undergone treatment for more than 30 days in the treatment group. The CKD grade was determined by calculating eGFR from creatinine level using Modification of Diet in Renal Disease (MDRD) GFR. The study was approved by the Seoul National University Boramae Medical Center Institutional Review Board (No. 10-2018-10/031), and requirement for consent was waived by virtue of the retrospective design. All clinical studies were conducted in accordance with the guidelines of the Declaration of Helsinki as amended in 2013.

### Clinical outcomes

The primary outcome was to clarify the all-cause mortality according to the TCO_2_ level, and the secondary outcome was to find a new development of ESRD that required dialysis and progression to mortality from various causes. We obtained mortality information from the national statistics office^17^, and ESRD incidence was defined as follows: patients recently undergoing hemodialysis were extracted from the electronic medical records using the clinical data warehouse in our study centers. Patients who had started hemodialysis therapy were identified by the combination of operational definitions as follows: the occurrence of a new claim for the payment code of hemodialysis by screening ICD-10 codes, and vascular access operations; incident dialysis was also identified by the first date of when dialysis was suggested.

### Statistical analysis

We used the chi-squared test for categorical variables and the Student’s t-test for continuous variables to compare the demographics. Categorical variables were reported as the percentage of all patients, and continuous variables as the mean ± standard deviation. The multivariate Cox proportional hazards model was used to calculate the hazard ratio (HR) with a 95% CI for all-cause mortality and renal outcomes. Kaplan–Meier curves and the multivariate Cox proportional hazard models were used to compare the outcomes between the Low TCO_2_ group and control groups. We included covariates, such as TCO_2_ values, age, sex, history of hypertension, diabetes mellitus, MDRD-GFR, and SB usage, in the final model using multivariate Cox regression analysis.

To minimize the bias of this retrospective observational study, we conducted an IV analysis^18–21^. The IV in this study was regional classification of proportion of patients with TCO_2_ < 22 mEq/L. We used the IV to independently associate the effects of low TCO_2_ level and clinical outcomes. All statistical analyses were performed using R version 3.5.0^22^ (Comprehensive R Archive Network: http://cran.r-project.org) and SPSS version 22.0 (IBM Corp., Armonk, NY, USA). The maps in supplementary figures was generated that outlines of administrative districts were obtained from Statistical Geographic Information Service of Statistics Korea (URL, https://sgis.kostat.go.kr/jsp/english/index.jsp). Maps were produced with R in conjunction with the software packages ggmap, maptools, rgdal, abd ggplot2^23–26^. In all analyses, *P* < 0.05 was considered statistically significant.

## Results

### Baseline characteristics and demographics

The baseline characteristics and demographics of the study population divided according to the CKD stage are shown in Table 1. The mean age of the study subjects was 54 years, and 48.9% were male. Hypertension and diabetes were found in 29.2% and 20.9% of patients, respectively. The mean eGFR was 70.5 mL/min/1.73 m^2^ and the TCO_2_ was 26.2 mEq/L. CKD stage 2 accounted for 44.8% of all patients, and about 10% of patients were in advanced stage with CKD stage 4–5. As CKD progressed, a decrease in TCO_2_ levels was observed, and the use of sodium bicarbonate, RAAS blockade, or diuretics was more frequent.

**Table 1:** Baseline characteristics and demographics according to the stage of chronic kidney disease.

	Total (n = 42,231)	CKD 1 (n = 9,641)	CKD 2 (n = 18,929)	CKD 3 (n = 9,446)	CKD 4 (n = 2,621)	CKD 5 (n = 1,594)	*P* value
Male (n, %)	20,681 (48.9)	4181 (43.4)	8607 (45.5)	5698 (60.3)	1373 (52.4)	822 (51.6)	< 0.0001
Age (years)	54.1 ± 16.5	42.5 ± 16.3	55.1 ± 14.8	62.7 ± 13.9	59.2 ± 15.1	53.0 ± 13.8	< 0.0001
Hypertension (n, %)	12,364 (29.2)	1385 (14.4)	4640 (24.5)	4218 (44.7)	1345 (51.3)	776 (48.7)	< 0.0001
Diabetes (n, %)	8849 (20.9)	1290 (13.4)	3819 (20.2)	2584 (27.4)	787 (30.0)	369 (23.1)	< 0.0001
Smoking (n, %)	2861 (6.8)	593 (18.0)	1171 (14.4)	750 (15.1)	221 (14.9)	126 (11.1)	< 0.0001
Systolic BP (mmHg)	130.8 ± 20.7	126.0 ± 18.8	129.3 ± 19.1	133.1 ± 21.7	137.3 ± 23.6	137.1 ± 22.8	< 0.0001
MDRD-GFR (mL/min/1.73 m^2^)	70.5 ± 29.4	107.4 ± 20.3	75.0 ± 8.1	47.2 ± 8.6	23.0 ± 4.3	9.1 ± 3.3	< 0.0001
Serum creatinine (mg/dL)	1.3 ± 1.3	0.8 ± 0.1	1.0 ± 0.2	1.5 ± 0.3	2.8 ± 0.6	7.0 ± 3.1	< 0.0001
Uric acid (mg/dL)	5.7 ± 2.0	4.8 ± 1.5	5.2 ± 1.5	6.7 ± 1.9	7.7 ± 2.1	7.8 ± 2.4	< 0.0001
Na (mEq/L)	140.1 ± 3.1	139.8 ± 3.2	140.4 ± 2.7	140.0 ± 3.1	139.4 ± 3.5	138.2 ± 4.1	< 0.0001
K (mEq/L)	4.4 ± 0.6	4.2 ± 0.4	4.3 ± 0.4	4.5 ± 0.6	4.9 ± 0.8	5.0 ± 0.9	< 0.0001
TCO_2_ (mEq/L)	26.2 ± 3.7	26.8 ± 3.1	27.1 ± 3.1	25.6 ± 3.6	22.5 ± 3.9	21.5 ± 5.0	< 0.0001
Usage of sodium bicarbonate	2659 (6.3)	153 (1.6)	511 (2.7)	971 (10.3)	686 (26.2)	338 (21.2)	< 0.0001
Usage of RAAS blockade	6010 (14.2)	1124 (11.7)	2479 (13.1)	1642 (17.3)	505 (19.3)	260 (16.3)	< 0.0001
Usage of diuretics							< 0.0001
Diuretics group 1	521 (1.2)	112 (1.2)	253 (1.3)	123 (1.3)	25 (1.0)	8 (0.5)	
Diuretics group 2	2318 (5.5)	288 (3.0)	800 (4.2)	785 (8.3)	308 (11.8)	137 (8.6)	
Crude ESRD rate (n, %)	3607 (8.5)	207 (2.1)	529 (2.8)	1,171 (12.4)	985 (36.6)	742 (46.5)	< 0.0001
Crude mortality rate (n, %)	5157 (12.2)	423 (4.4)	1634 (8.6)	1,953 (20.7)	692 (26.4)	455 (28.5)	< 0.0001

The data are expressed as the proportion (%), mean ± SD or median (IQR).

*BP* blood pressure, *ESRD* end-stage renal disease, *GFR* glomerular filtration rate, *MDRD* modification of diet in renal disease, *TCO*_*2*_ total CO_2_.

RAAS blockade included Angiotensin-converting-enzyme inhibitors ( ACE inhibitors*,* ACEi) and Angiotensin II receptor blockers (*ARBs*).

Diuretics group 1 included acetazolamide, spironolactone, amiloride, and triamterene.

Diuretics group 2 included furosemide, torasemide, thiazide, indapamide, and metolazone.

Meanwhile, the serum TCO_2_ level was < 22 mEq/L in 10.1% of all patients, and we graphically depicted the proportion of patients with a TCO_2_ < 22 mEq/L by the patient’s residential location (Supplementary Information Fig. S1). When we divided patients into four groups based on the proportion of serum TCO_2_ concentration < 22 mEq/L by residential area, we found that hypertension and smoking history were the highest in the 4^th^ group (Supplementary Information Table S1). The first group comprised patients who were older, had a higher prevalence of diabetes mellitus, and had the best baseline renal function. The fourth group had the highest rate of low TCO_2_ level (*P* < 0.001).

### Association between low TCO_2_ and patient mortality

**Figure 2:**
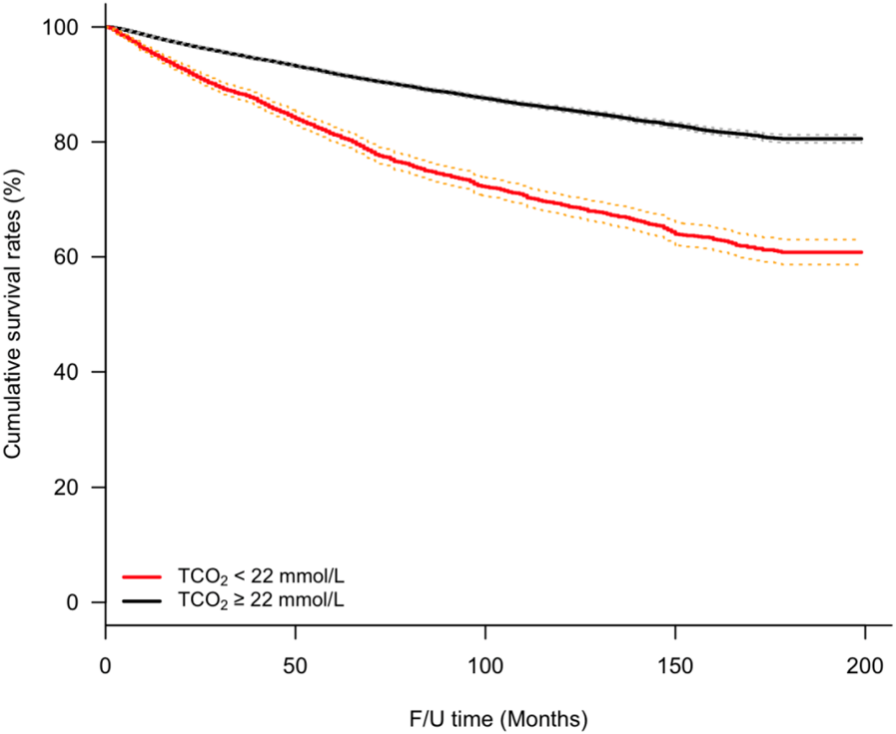
Survival analysis for all-cause mortality in the study cohort. Crude mortality rate was 12.2% during the median 77.0 months of follow-up, and Kaplan–Meier curve showed that Low TCO_2_ group had higher mortality, compared to normal TCO_2_ group.

During the median 77.0-month follow-up period, the crude mortality rate was 12.2%. According to the Kaplan–Meier curve (Fig. 2), patients with TCO_2_ < 22 mEq/L had a higher risk of mortality (HR 0.899; 95% CI 0.893–0.905; *P* < 0.001) than those in the other groups. The Cox-proportional hazard regression model adjusted for sex, age, hypertension, diabetes mellitus, and alkali supplementation showed that low TCO_2_ concentration increased the risk for mortality (model 3, Table 2). After adding of initial eGFR levels for adjustment, the association between low TCO_2_ concentration and mortality remained consistently significant (model 5). These results were consistently significant even when the use of RAAS blockades and diuretics were additionally adjusted (model 6). In the IV analysis, low TCO_2_ concentration also increased the mortality risk. When adjusted for baseline renal function, alkali supplementation, RAAS blockade, and diuretics, the mortality risk reduced by approximately 44% with each 1 mEq/L increase in serum TCO_2_ concentration (model 6; 95% CI 0.485–0.644; *P* < 0.001).

**Table 2:** Cox regression analysis for the risk of mortality.

Model	Cox regression analysis			Instrumental variable analysis		
	HR	95% CI	*P*-value	HR	95% CI	*P* value
1	0.899	(0.893, 0.905)	< 0.0001	0.554	(0.484, 0.635)	< 0.0001
2	0.912	(0.906, 0.918)	< 0.0001	0.486	(0.422, 0.559)	< 0.0001
3	0.912	(0.906, 0.917)	< 0.0001	0.485	(0.421, 0.559)	< 0.0001
4	0.939	(0.933, 0.946)	< 0.0001	0.559	(0.485, 0.643)	< 0.0001
5	0.938	(0.931, 0.945)	< 0.0001	0.559	(0.485, 0.644)	< 0.0001
6	0.942	(0.935, 0.949)	< 0.0001	0.559	(0.485, 0.644)	< 0.0001

Model 1: TCO_2_ only.

Model 2: Model 1 + sex, age, history of hypertension and diabetes.

Model 3: Model 2 + usage of sodium bicarbonate.

Model 4: Model 2 + baseline kidney function as MDRD-GFR.

Model 5: Model 4 + usage of sodium bicarbonate.

Model 6: Model 5 + RAAS blockades + Diuretics group.

*CI* confidence interval, *ESRD* end-stage renal disease, *GFR* glomerular filtration rate, *HR* hazard ratio, *MDRD* modification of diet in renal disease, *TCO*_*2*_ total CO_2_.

**Figure 3:**
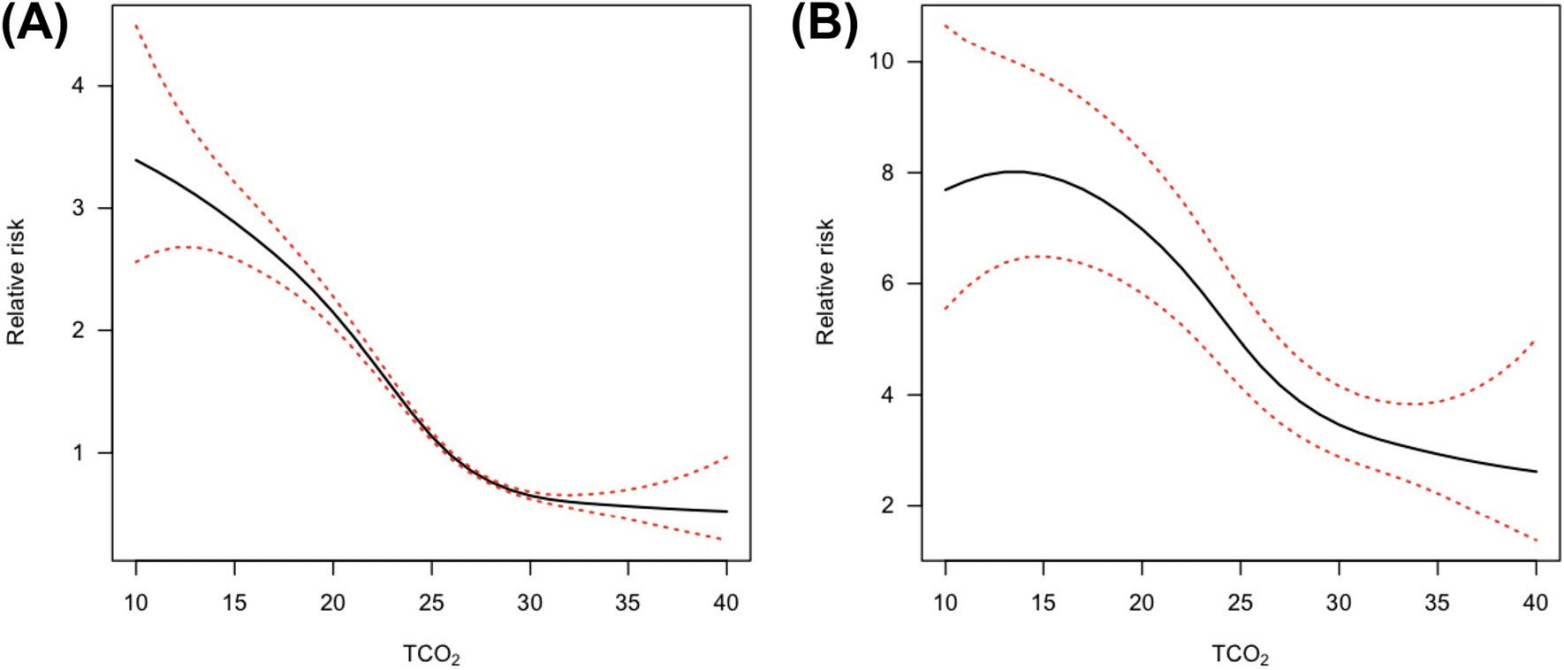
Non-linear association between the TCO_2_ levels and the risk of patient death. (**A**) Univariate analysis (**B**) Multivariate analysis. These graphs were adjusted for TCO_2_ level (as continuous variable), age, sex, history of hypertension and diabetes, baseline kidney function (as continuous variable using MDRD-eGFR), usage of sodium bicarbonate, RAAS blockades and diuretics.

Next, the TCO_2_ level was set as a continuous variable and the correlation with the risk of death was visually shown (Fig. 3). As a result, it was confirmed that the lower the TCO_2_ level, the higher the risk of death (Fig. 3A). Even after adjusting for several variables including age, sex, hypertension, diabetes, baseline kidney function, usage of sodium bicarbonate, RAAS blockades and diuretics, a consistent pattern was shown (Fig. 3B).

It was also verified that these results were consistent in Table 3, which was expressed as a categorical variable. Based on the serum TCO_2_ concentration, patients were divided into three groups as follows: low, < 22 mEq/L; normal, 22–29 mEq/L; high, ≥ 30 mEq/L. Compared to the normal group, mortality risk was significantly higher in patients with serum TCO_2_ concentration below 22 mEq/L; however, the risk decreased when TCO_2_ concentrations were above 30 mEq/L. These results were significant even after adjusting for other variables.

**Table 3:** Cox regression analysis for the risk of mortality according to the TCO_2_ category groups.

Outcome	Model	Group	HR	95% CI	*P* value
Mortality	1	Low versus normal	2.231	(2.084, 2.389)	< 0.001
		High versus normal	0.623	(0.567, 0.685)	< 0.001
	2	Low versus normal	1.967	(1.836, 2.107)	< 0.001
		High versus normal	0.661	(0.601, 0.726)	< 0.001
	3	Low versus normal	1.472	(1.366, 1.585)	< 0.001
		High versus normal	0.715	(0.650, 0.786)	< 0.001
	4	Low versus normal	1.484	(1.377, 1.599)	< 0.001
		High versus normal	0.713	(0.649, 0.785)	< 0.001
	5	Low versus normal	1.481	(1.375, 1.595)	< 0.001
		High versus normal	0.713	(0.649, 0.784)	< 0.001

Model 1: TCO_2_ only.

Model 2: Model 1 + sex, age, history of hypertension and diabetes.

Model 3: Model 2 + baseline kidney function as MDRD-GFR.

Model 4: Model 3 + usage of sodium bicarbonate.

Model 5: Model 4 + RAAS blockades + Diuretics group.

Group definition: Low group, < 22 mEq/L versus Normal group, 22–29 mEq/L versus High group, ≥ 30 mEq/L.

*CI* confidence interval, *ESRD* end-stage renal disease, *GFR* glomerular filtration rate, *HR* hazard ratio, *MDRD* modification of diet in renal disease, *TCO*_*2*_ total CO_2_.

In the subgroup analysis by initial eGFR, mortality risk increased with lower serum TCO_2_ concentrations, regardless of initial GFR values (Table 4). These results were verified both in the Cox-proportional hazard regression model and on IV analysis. This also showed significant results when adjusting for underlying renal function, underlying disease, and medications taken, and the effect was more pronounced in patients with relatively good renal function with a baseline GFR of 60 mL/min/1.73 m^2^ or higher.

**Table 4:** Subgroup analysis for the risk of mortality according to the baseline GFR.

Subgroup	Model	Cox regression analysis			Instrumental variable analysis		
		HR	95% CI	*P*	HR	95% CI	*P*
GFR < 30 (n = 4215)	1	0.989	(0.976, 1.002)	0.089	0.823	(0.656, 1.031)	0.090
	2	0.988	(0.975, 1.001)	0.072	0.801	(0.625, 1.027)	0.081
	3	0.986	(0.973, 0.999)	0.036	0.778	(0.607, 0.998)	0.048
	4	0.981	(0.968, 0.994)	0.004	0.760	(0598, 0.966)	0.025
	5	0.996	(0.983, 1.009)	0.537	0.759	(0.606, 0.950)	0.016
	6	0.991	(0.977, 1.004)	0.173	0.746	(0.601, 0.926)	0.008
	7	0.993	(0.980, 1.007)	0.329	0.720	(0.582, 0.890)	0.002
30 ≤ GFR < 60 (n = 9446)	1	0.924	(0.913, 0.935)	< 0.001	0.769	(0.682, 0.867)	< 0.001
	2	0.926	(0.915, 0.937)	< 0.001	0.759	(0.672, 0.857)	< 0.001
	3	0.918	(0.907, 0.929)	< 0.001	0.688	(0.609, 0.776)	< 0.001
	4	0.915	(0.904, 0.927)	< 0.001	0.687	(0.609, 0.776)	< 0.001
	5	0.925	(0.914, 0.937)	< 0.001	0.662	(0.571, 0.766)	< 0.001
	6	0.923	(0.911, 0.935)	< 0.001	0.667	(0.577, 0.772)	< 0.001
	7	0.926	(0.914, 0.938)	< 0.001	0.615	(0.531, 0.711)	< 0.001
GFR ≥ 60 (n = 28,570)	1	0.924	(0.912, 0.936)	< 0.001	0.412	(0.221, 0.770)	0.005
	2	0.939	(0.927, 0.951)	< 0.001	0.390	(0.217, 0.700)	0.002
	3	0.934	(0.922, 0.946)	< 0.001	0.305	(0.170, 0.547)	< 0.001
	4	0.937	(0.925, 0.949)	< 0.001	0.326	(0.185, 0.573)	< 0.001
	5	0.936	(0.924, 0.948)	< 0.001	0.273	(0.136, 0.549)	< 0.001
	6	0.939	(0.927, 0.952)	< 0.001	0.296	(0.151, 0.580)	< 0.001
	7	0.942	(0.930, 0.954)	< 0.001	0.280	(0.145, 0.540)	< 0.001

Model 1: TCO_2_ only.

Model 2: Model 1 + history of hypertension and diabetes.

Model 3: Model 2 + sex and age.

Model 4: Model 3 + usage of sodium bicarbonate.

Model 5: Model 3 + baseline kidney function as MDRD-GFR.

Model 6: Model 5 + usage of sodium bicarbonate.

Model 7: Model 6 + RAAS blockades + Diuretics group.

*CI* confidence interval, *GFR* glomerular filtration rate, *HR* hazard ratio, *TCO*_*2*_ total CO_2_.

### Association between low TCO_2_ and the risk of progression to ESRD

The risk of progression to ESRD steadily increased over time and occurred in 8.5% of patients during the follow-up period. Patients with TCO_2_ < 22 mEq/L showed higher risks of progression to ESRD (HR 0.844; 95% CI 0.838–0.850; P < 0.001) than the other groups in univariate analysis (Supplementary Information Fig. S2, Table S2). However, the protective effect was the lowest in final model 6, which was adjusted for baseline renal function and medications. These results are consistent with the IV analysis. After adjusting for baseline renal function and medications taken, there was no association between serum TCO_2_ concentration and progression to ESRD (model 6; HR 1.057, 95% CI 0.892–1.251, *P* = 0.5234).

The subgroup analysis of the relationship between the TCO_2_ level and ESRD progression risk by TCO_2_ level groups is also presented in Supplementary Information Fig. S3 and Table S3. In subgroup IV analysis by initial eGFR, serum TCO_2_ concentration did not affect ESRD progression, regardless of eGFR (Supplementary Information Table S4).

## Discussion

This study is the first population-scale study to show that low TCO_2_ level affected clinical outcomes in an East-Asian population. The main objective of this study was to determine whether the single measurement of TCO_2_ level affected the clinical outcomes in patients with ambulatory care in out-patients clinic, whose diet patterns were thought to be less acidic than that of their Western counterparts. According to the Korea National Health and Nutrition Examination Survey data^27,28^, regional differences were noted in meat intake between urban and rural areas. These differences in animal-based acidic diet pattern were likely related to the rate of dietary acid loading, causing eubicarbonatic H^+^ retention without overt metabolic acidosis^13–15^. We used the fraction of low TCO_2_ levels (< 22 mEq/L) by the residence area of participants as the IV to determine the independent relationship between TCO_2_ level and clinical outcomes. To be selected as an IV, several important assumptions must be met^19,20^. First, the exposure values should have definite predictive associations with the causal inference for IV. This IV should not be directly related to the outcome to be predicted. The four groups in Supplementary Information Table S1 did not differ significantly in terms of renal function by eGFR or other baseline characteristics, but only in terms of the proportion of patients with low TCO_2_ level.

Final Cox regression models and IV analysis including covariates of initial eGFR and information on the use of medications such as SB, RAAS blockade, and diuretics, showed that higher TCO_2_ concentrations were protective in patients at risk of all-cause mortality, but not for preventing progression to ESRD (Tables 2 and Supplementary Information Table S2). Serum bicarbonate concentrations were not very low in most of the patients in this study; only 10% of the cohort (4,242/42,331) had a serum bicarbonate level of < 22 mEq/L. Our results are consistent with those of previous studies which showed that long-term prognosis improved in patients with preserved renal function when serum bicarbonate levels were at the upper limit of the normal range^6^. Notably, in this study, the authors noted that hypobicarbonatemia (identified by low TCO_2_ levels) may not indicate metabolic acidosis, and this is a possible limitation of the study. The identification of acid–base status based on TCO_2_ as bicarbonate level may have led to misclassification because low TCO_2_ might be due to chronic respiratory or mixed acid–base disorders. These misclassifications could be seen in patients with ESRD receiving hemodialysis^29^ and in patients with acute renal failure requiring continuous renal replacement therapy^30^. However, in patients with preserved renal function in an outpatient setting, TCO_2_ is considered effective for predicting serum bicarbonate level^31^. Thus, we believe that the low TCO_2_ group in this study mainly reflects hypobicarbonatemia, irrespective of pH status. A recent study by Hirai et al. showed that serum TCO_2_ correlated with bicarbonate concentration in Japanese patients with pre-dialysis CKD (sensitivity 91.7%, specificity 73.4%)^32^, supporting our results.

In terms of ESRD development, there was significance in model 3 without eGFR as a covariate; however, the protective effect of high TCO_2_ disappeared when baseline eGFR was considered (Supplementary Information Table S2, model 4 to 6). Recent studies on early CKD (CKD2-3a) patients reveal that H^+^ retention is first seen, even if low TCO_2_ is not present yet as eubicarbonatemia. This H^+^ retention itself affects CKD progression, which can be diagnosed by hypocitrauria^13^ and urine ammonium^15^. In addition, low TCO_2_ itself is a highly independent risk factor, which is more pronounced in early CKD and above (Table S3), and in advanced CKD (GFR 30 mL/min/1.73 m^2^ and below), the influence on the ESRD of various factors other than TCO_2_ level itself is much greater (e.g., vascular calcification, cardiovascular disease, and commodities). In the results of our study, the final Cox model and IV analysis were inconclusive in terms of ESRD incidence as there were limitations due to the retrospective study design, and heterogeneity of the operational definition of ESRD was also limitation. Another possible rationale for hypobicarbonatemia being associated only with mortality but not with ESRD incidence in our study involves systemic compensation capacity. A recent study in patients with CKD found that hypobicarbonatemia was involved in CKD progression only when pH reached acidemia (pH < 7.32)^33^. The acid–base status (using gas analysis of blood pH) on the clinical outcome of hypobicarbonatemia was not considered in existing studies^4,6,11^. According to researchers at the University of Osaka, in a study of 1,058 patients with CKD (eGFR < 60 mL/min), TCO_2_ level and pH were measured simultaneously through venous blood gas analysis (VBGA). This study showed that the strength of this association is significantly changed by acidemia. Hypobicarbonatemia is associated with an increased risk of ESRD only when pH is low (pH < 7.32) but not when pH is normal. This association suggests that the compensatory reservoir might be important in terms of CKD prognosis^15^. In our study, patients with CKD 1,2 (eGFR > 60 mL/min) were 67% of the total. These patients are likely to have a normal pH. Results from the Osaka researchers also found that 40% of the group with bicarbonate levels under 21.5 mEq/L did not present acidemia as a result of systemic compensation^33^. In addition, in a prospective cohort study of an elderly population without CKD, similar to our results, 60% of the group with low bicarbonate levels did not have acidemia^34^. This study showed a correlation between low bicarbonatemia levels and high mortality, regardless of pH, and the modification of the effects by pH did not affect this association, which supports our findings. These findings suggest that combining information on pH with bicarbonate levels, especially to improve renal outcome, helps identify patients at high risk of ESRD. In addition, needless alkali treatments could be reduced in non-acidic hypobicarbonatemic patients^15, 33^. Further studies may identify the need for clinicians to further improve non-acidicidc hypobicarbonatemic or eubicarbonatic H^+^ retension in early CKD patients for slowing CKD progression. Treatment and consequent improvement of acidosis in CKD patients are expected to improve the clinical outcomes; however, the optimal interventional methods and population remain inconclusive, and The cautions is needen for the replacement of alkali using SB which is associated with complications, such as deterioration of sodium retention, edema, and heart failure aggravation.

In conclusion, low TCO_2_ levels were significantly associated with mortality, but not with progression to ESRD in IV analysis using regional classification by proportion of low TCO_2_ as an IV. Further studies, for a prospective interventional study on a nation-wide scale for the effects of alkali therapy on CKD progression in patients with eubicarbonatemia with early CKD and explanations for possible biologic mechanisms, in Korean CKD patients are needed.

## Supplementary Information

Supplementary Information.

## Data Availability

All data associated with this study are available in the main text or the supplementary materials following reasonable request.
