# Supplementary material for: Low serum total CO2 and its association with mortality in patients being followed up in the nephrology outpatients clinic
Source: Sci Rep. 2021 Jan 18;11:1711. doi: 10.1038/s41598-021-81332-2 (PMC7814051; doi:10.1038/s41598-021-81332-2)
Supplement: Supplementary file 1 — Supplementary Information. [file 41598_2021_81332_MOESM1_ESM.pdf]

[Title page]

Low Serum Total CO_2 and its Association with Mortality in Patients Being Followed up in the Nephrology Outpatients Clinic

Kyung Don Yoo[1*], Jung Nam An[2*], Yong Chul Kim[3], Jeonghwan Lee[4], Kwon-Wook Joo[3,5], Yun Kyu Oh[4,5], Yon Su Kim[3,5], Chun Soo Lim[4,5], Sohee Oh[6¶], Jung Pyo Lee[4,5¶]

[1]Department of Internal Medicine, Ulsan University Hospital, University of Ulsan College of Medicine, Ulsan, Korea

[2]Department of Internal Medicine, Hallym University Sacred Heart Hospital, Anyang, Gyeonggi-do, Korea

[3]Department of Internal Medicine, Seoul National University Hospital, Seoul, Korea

[4]Department of Internal Medicine, Seoul National University Boramae Medical Center, Seoul, Korea

[5]Department of Internal Medicine, Seoul National University College of Medicine, Seoul, Korea

[6]Department of Biostatistics, Seoul National University Boramae Medical Center, Seoul, Korea

[*]Yoo KD and An JN contributed equally to this research.

¶**Corresponding Authors**

Jung Pyo Lee, MD, PhD

Associate Professor

Department of Internal Medicine, Seoul National University College of Medicine and Seoul National University Boramae Medical Center

20, Boramae-ro 5-gil, Dongjak-gu, Seoul, 07061 (Republic of Korea)

Tel. +82 2 870 3206

Fax +82 2 831 2826

Email: nephrolee@gmail.com

Sohee Oh, PhD

Assistant Professor

Department of Biostatistics, Seoul National University Boramae Medical Center

20, Boramae-ro 5-gil, Dongjak-gu, Seoul, 07061 (Republic of Korea)

Tel. +82 2 870 3855

Fax +82 2 831 2826

Email: oh.sohee@gmail.com

Jung Pyo Lee and Sohee Oh contributed equally to this research as corresponding authors.

Supplementary Information

Supplement figure legends

Figure S1. Graphical presentation of the proportion of patients with TCO_2 < 22 mEq/L

(A) Metropolitan area of South Korea (B) Local province around Seoul city (C) Seoul

The maps in these figures were generated that outlines of administrative districts were obtained from Statistical Geographic Information Service of Statistics Korea (URL, https://sgis.kostat.go.kr/jsp/english/index.jsp). Maps were produced with the R in conjunction with software packages ggmap, maptools, rgdal, abd ggplot2.[1-4]

Figure S2. Survival analysis for progression to ESRD in the study cohort

The incident ESRD occurred in 8.5% of the patients during the median 77.0 months of follow-up, and Kaplan-Meier curve showed that low TCO_2 group had higher event rate, compared to normal TCO_2 group.

Figure S3. Non-linear association between the TCO_2 levels and the risk of progression to ESRD

(A) Univariate analysis (B) Multivariate analysis. These graphs were adjusted for TCO_2 level (as continuous variable), age, sex, history of hypertension and diabetes, baseline kidney function (as continuous variable using MDRD-eGFR), usage of sodium bicarbonate, RAAS blockades and diuretics.

Supplementary tables

Table S1. Baseline characteristics and demographics according to the proportion of metabolic acidosis by residental area

	Proportion of metabolic acidosis (TCO_2 < 22 mEq/L)					*P*-value
	Total (n=42,231)	< 8.8% (n=10,983)	8.8 ≤ < 10.0% (n=10,271)	10.0 ≤ < 11.3% (n=10,558)	≥ 11.3% (n=10,419)	
**Male (n, %)**	20,681 (48.9)	5,305 (48.3)	4,923 (47.9)	5,268 (49.9)	5,190 (49.8)	0.0055
**Age (years)**	54.16 ± 16.56	54.8 ± 16.4	54.0 ± 16.6	53.6 ± 16.4	54.3 ± 16.8	< 0.0001
**Hypertension (n, %)**	12,364 (29.2)	3,085 (28.1)	2,868 (27.9)	3,029 (28.7)	3,382 (32.5)	< 0.0001
**Diabetes (n, %)**	8,849 (20.9)	2,515 (22.9)	2,294 (22.3)	2,167 (20.5)	1,873 (18.0)	< 0.0001
**Smoking (n, %)**	2,861 (6.8)	678 (6.1)	685 (6.6)	748 (7.0)	750 (7.1)	< 0.0001
**Systolic BP (mmHg)**	130.8 ± 20.7	130.0 ± 19.6	130.0 ± 20.4	130.9 ± 20.6	132.3 ± 21.9	< 0.0001
**MDRD-GFR (ml/min/1.73 m^2)**	70.5 ± 29.4	71.4 ± 27.8	70.8 ± 28.2	69.4 ± 30.2	70.4 ± 31.4	< 0.0001
**Serum creatinine (mg/dL)**	1.3 ± 1.3	1.30 ± 1.25	1.35 ± 1.37	1.45 ± 1.52	1.41 ± 1.39	< 0.0001
**Uric acid (mg/dL)**	5.68 ± 1.96	5.62 ± 1.91	5.63 ± 1.94	5.73 ± 1.98	5.75 ± 2.00	< 0.0001
**Na (mmol/L)**	140.1 ± 3.1	140.2 ± 3.2	140.2 ± 2.9	140.0 ± 3.1	139.9 ± 3.13	< 0.0001
**K (mmol/L)**	4.38 ± 0.55	4.36 ± 0.52	4.37 ± 0.54	4.40 ± 0.57	4.39 ± 0.57	< 0.0001
**TCO_2 (mmol/L)**	26.18 ± 3.69	26.42 ± 3.53	26.30 ± 3.69	26.13 ± 3.73	25.85 ± 3.77	< 0.0001
**Crude ESRD rate (n, %)**	3,607 (8.54)	865 (7.9)	846 (8.2)	1,044 (9.9)	852 (8.2)	< 0.0001
**Crude Mortality rate (n, %)**	5,157 (12.21)	1,284 (11.7)	1,213 (11.8)	1,364 (12.9)	1,296 (12.4)	0.021

The data are expressed as the proportion (%), mean ± SD or median (IQR).

Abbreviations: BP, blood pressure; ESRD, end-stage renal disease; GFR, glomerular filtration rate; MDRD, Modification of Diet in Renal Disease; TCO_2, total CO_2

Table S2. Cox regression analysis for the risk of progression to ESRD

	Cox regression analysis			Instrumental variable analysis		
**Model**	**HR**	**95% CI**	***P*-value**	**HR**	**95% CI**	***P*-value**
1	0.844	(0.838, 0.850)	<0.0001	0.59	(0.500, 0.695)	<0.0001
2	0.869	(0.863, 0.875)	<0.0001	0.634	(0.536, 0.750)	<0.0001
3	0.900	(0.893, 0.907)	<0.0001	0.685	(0.578, 0.812)	<0.0001
4	0.975	(0.967, 0.983)	<0.0001	1.039	(0.875, 1.235)	0.6619
5	0.992	(0.984, 1.000)	0.0505	1.047	(0.880, 1.246)	0.6021
6	0.992	(0.984, 0.999)	0.0483	1.057	(0.892, 1.251)	0.5234

Model 1: TCO_2 only
Model 2: Model 1 + sex, age, history of hypertension and diabetes
Model 3: Model 2 + usage of sodium bicarbonate
Model 4: Model 2 + baseline kidney function as MDRD-GFR
Model 5: Model 4 + usage of sodium bicarbonate
Model 6: Model 5 + RAAS blockades + Diuretics group

Abbreviations: CI, confidence interval; ESRD, end-stage renal disease; GFR, glomerular filtration rate; HR, hazard ratio; MDRD, Modification of Diet in Renal Disease; TCO_2, total CO_2

Table S3. Cox regression analysis for the risk of ESRD-events according to the TCO_2 category groups

Outcome	Model	Group	HR	95% CI	*P*-value
ESRD	1	Low vs. Normal	3.868	(3.596, 4.161)	<0.001
		High vs. Normal	0.463	(0.405, 0.529)	<0.001
	2	Low vs. Normal	3.081	(2.863, 3.315)	<0.001
		High vs. Normal	0.514	(0.450, 0.587)	<0.001
	3	Low vs. Normal	1.130	(1.043, 1.225)	0.003
		High vs. Normal	0.699	(0.611, 0.799)	<0.001
	4	Low vs. Normal	0.988	(0.912, 1.071)	0.775
		High vs. Normal	0.770	(0.673, 0.882)	<0.001
	5	Low vs. Normal	0.984	(0.908, 1.067)	0.700
		High vs. Normal	0.765	(0.669, 0.876)	<0.001

Model 1: TCO_2 only
Model 2: Model 1 + sex, age, history of hypertension and diabetes
Model 3: Model 2 + baseline kidney function as MDRD-GFR
Model 4: Model 3 + usage of sodium bicarbonate
Model 5: Model 4 + RAAS blockades + Diuretics group

Group definition: Low group, <22 mmol/L *vs.* Normal group, 22-29 mmol/L *vs.* High group, ≥ 30 mmol/L

Abbreviations: CI, confidence interval; ESRD, end-stage renal disease; GFR, glomerular filtration rate; HR, hazard ratio; MDRD, Modification of Diet in Renal Disease; TCO_2, total CO_2

Table S4. Subgroup analysis for the progression to ESRD according to the baseline GFR

		Cox regression analysis			Instrumental variable analysis		
**Subgroup**	**Model**	**HR**	**95% CI**	***P***	**HR**	**95% CI**	***P***
**GFR < 30 (n=4,215)**	1	0.994	(0.983, 1.005)	0.259	1.111	(0.936, 1.318)	0.230
	2	0.998	(0.987, 1.009)	0.695	1.200	(0.999, 1.441)	0.051
	3	0.998	(0.987, 1.009)	0.736	1.211	(1.008, 1.455)	0.041
	4	1.006	(0.995, 1.017)	0.323	1.218	(1.008, 1.472)	0.041
	5	1.006	(0.995, 1.017)	0.296	1.156	(0.985, 1.358)	0.077
	6	1.016	(1.005, 1.027)	0.005	1.162	(0.985, 1.370)	0.075
	7	1.015	(1.004, 1.026)	0.009	1.151	(0.979, 1.354)	0.089
**30≤GFR<60 (n=9,446)**	1	0.928	(0.914, 0.942)	<0.001	0.884	(0.750, 1.041)	0.139
	2	0.934	(0.919, 0.949)	<0.001	0.863	(0.732, 1.018)	0.081
	3	0.940	(0.925, 0.955)	<0.001	0.903	(0.765, 1.065)	0.225
	4	0.969	(0.954, 0.984)	<0.001	0.967	(0.821, 1.138)	0.685
	5	0.965	(0.949, 0.981)	<0.001	0.965	(0.782, 1.189)	0.736
	6	0.987	(0.971, 1.003)	0.112	1.014	(0.827, 1.244)	0.895
	7	0.987	(0.971, 1.003)	0.103	1.021	(0.835, 1.249)	0.837
**GFR ≥ 60 (n=28,570)**	1	0.894	(0.875, 0.913)	<0.001	1.348	(0.420, 4.328)	0.616
	2	0.922	(0.903, 0.941)	<0.001	0.841	(0.284, 2.489)	0.755
	3	0.920	(0.902, 0.940)	<0.001	0.993	(0.330, 2.990)	0.990
	4	0.954	(0.935, 0.973)	<0.001	0.963	(0.319, 2.901)	0.946
	5	0.920	(0.901, 0.939)	<0.001	0.977	(0.259, 3.685)	0.972
	6	0.954	(0.935, 0.973)	<0.001	0.976	(0.258, 3.700)	0.972
	7	0.954	(0.935, 0.973)	<0.001	1.219	(0.334, 4.450)	0.764

Model 1: TCO_2 only
Model 2: Model 1 + history of hypertension and diabetes
Model 3: Model 2 + sex and age
Model 4: Model 3 + usage of sodium bicarbonate
Model 5: Model 3 + baseline kidney function as MDRD-GFR
Model 6: Model 5 + usage of sodium bicarbonate
Model 7: Model 6 + RAAS blockades + Diuretics group

Abbreviations: CI, confidence interval; ESRD, end-stage renal disease; GFR, glomerular filtration rate; HR, hazard ratio; TCO_2, total CO_2

REFERENCE

1. Bivand, R. & Lewin-Koh, N. maptools: Tools for reading and handling spatial objects, 2014. *R package version 0.8-29* (2015).
2. Bivand, R., Keitt, T., Rowlingson, B. & Pebesma, E. rgdal: Bindings for the geospatial data abstraction library. *R package version* **1**(2016).
3. Wickham, H. *ggplot2: elegant graphics for data analysis*, (springer, 2016).
4. Kahle, D. & Wickham, H. ggmap: Spatial Visualization with ggplot2. *The R Journal* **5**, 144-161 (2013).

Fig. S1A

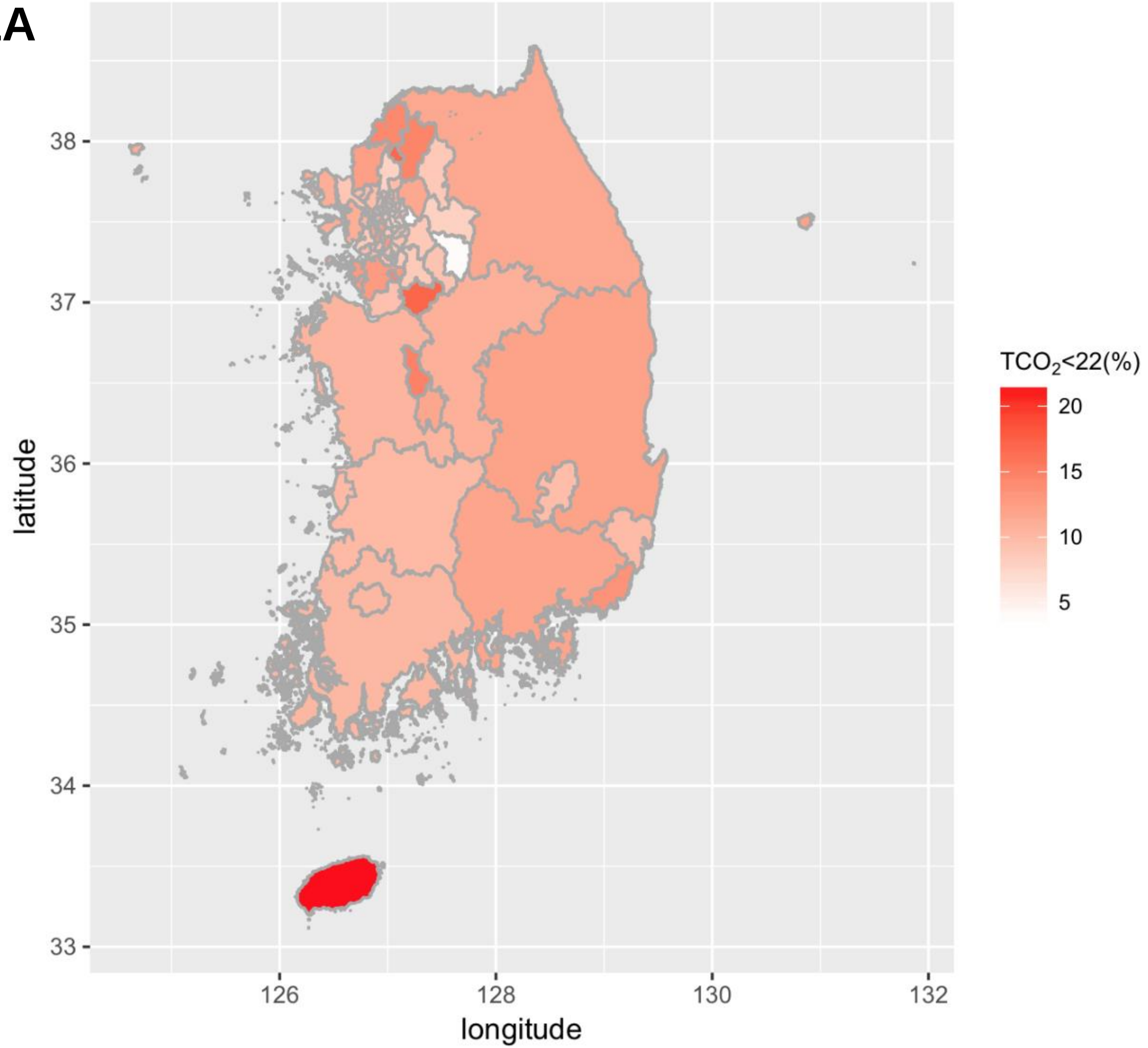

TCO$_2$<22(%)
20
15
10
5
38
37
36
35
34
33
latitude
126
128
130
132
longitude

Fig. S1B

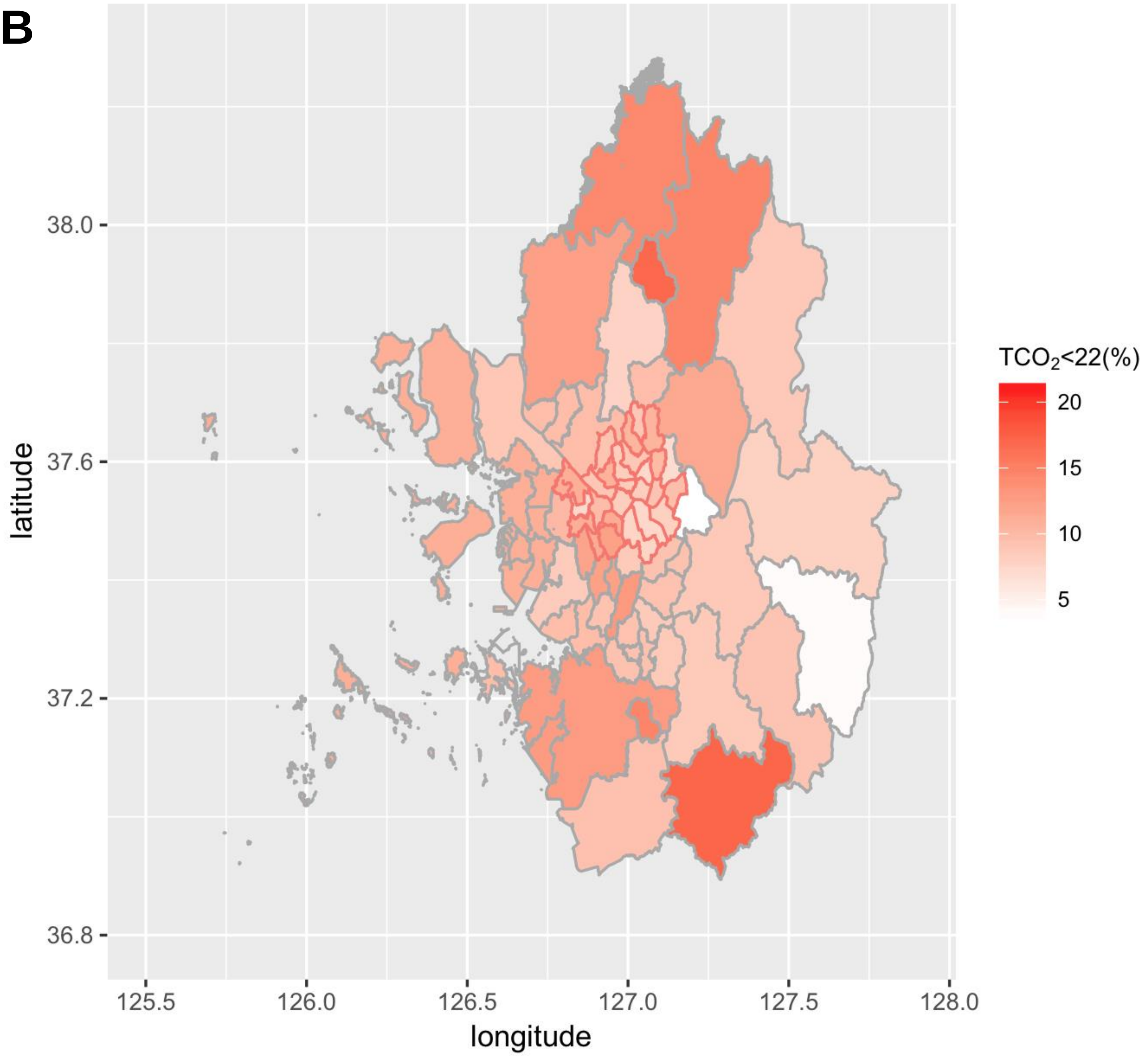

38.0
37.6
37.2
36.8
latitude
125.5
126.0
126.5
127.0
127.5
128.0
longitude
TCO_2<22(%)
20
15
10
5

Fig. S1C

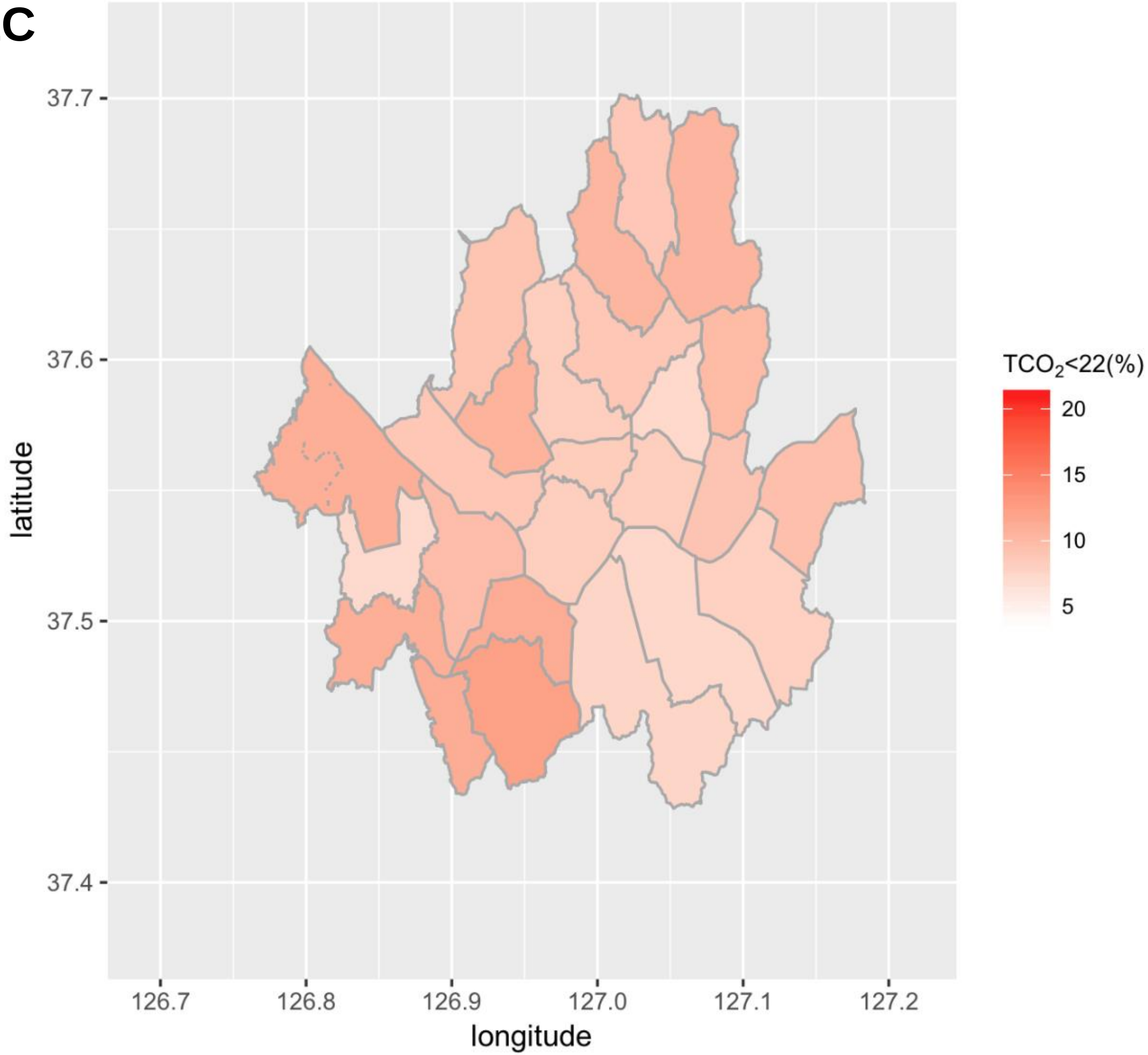
37.7
37.6
37.5
37.4
latitude
126.7
126.8
126.9
127.0
127.1
127.2
longitude
TCO2<22(%)
20
15
10
5

Fig. S2

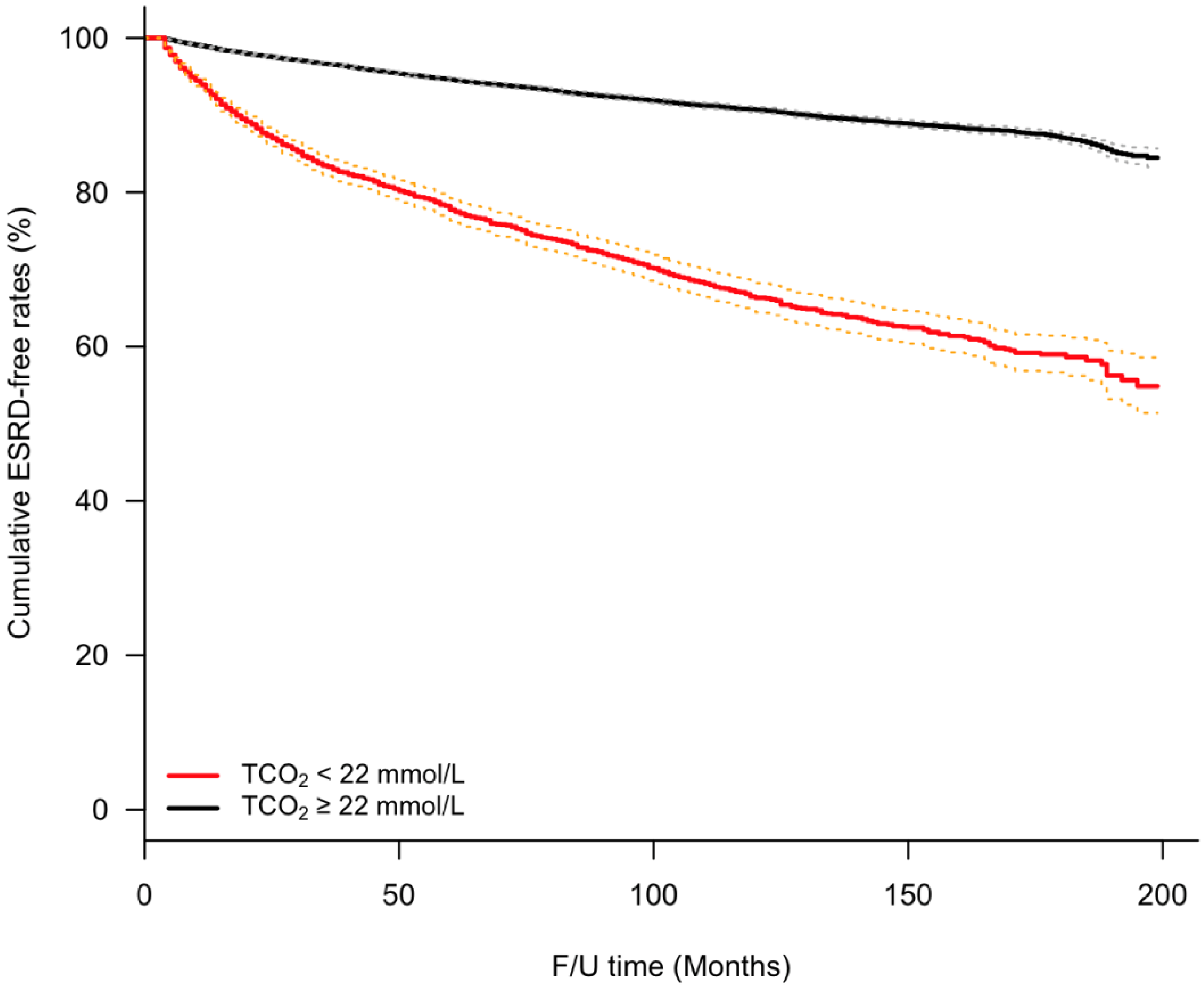

100
80
60
40
20
0
Cumulative ESRD-free rates (%)
0
50
100
150
200
F/U time (Months)
TCO2 < 22 mmol/L
TCO2 ≥ 22 mmol/L

Fig. S3

(A)

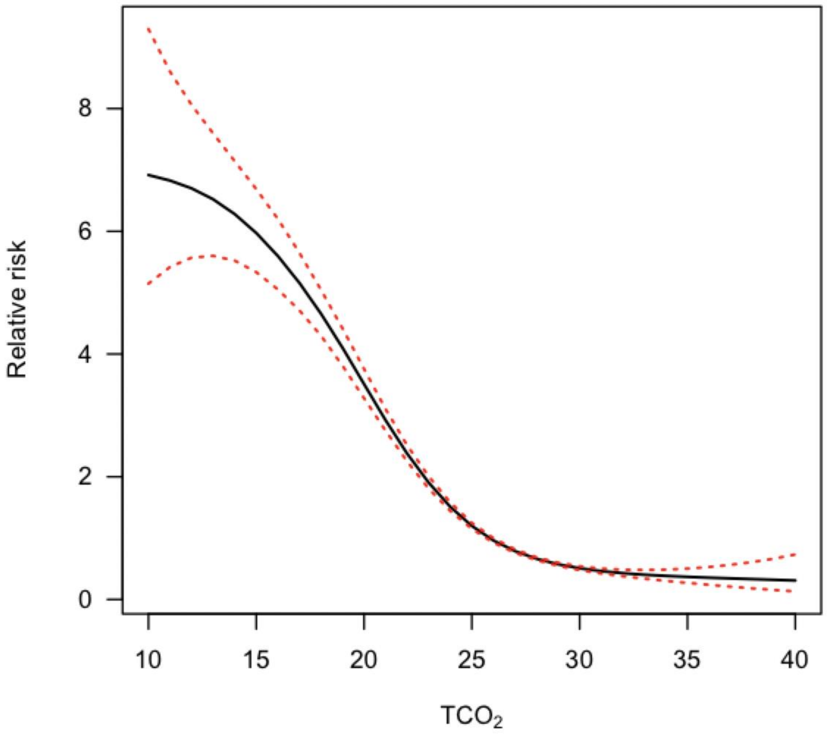

Relative risk
0
2
4
6
8
10
15
20
25
30
35
40
TCO2

(B)

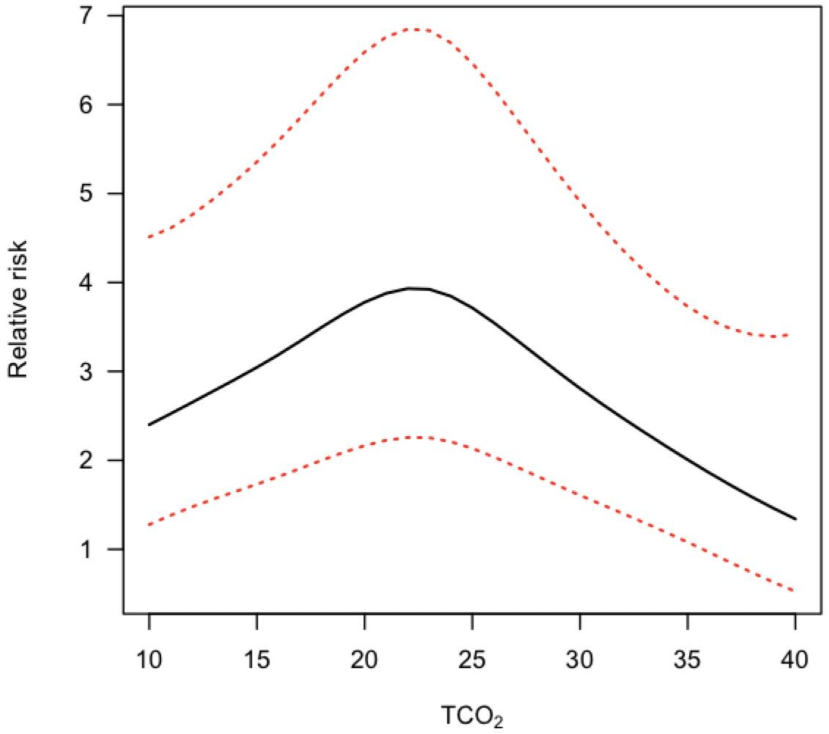

Relative risk
1
2
3
4
5
6
7
10
15
20
25
30
35
40
TCO2
